# Recurrent Anti-AMPA Receptor Limbic Encephalitis: A Case Report and Literature Review

**DOI:** 10.3389/fneur.2021.735983

**Published:** 2021-12-06

**Authors:** Yuanyuan Fang, Dengji Pan, Hao Huang

**Affiliations:** Department of Neurology, Tongji Medical College, Tongji Hospital, Huazhong University of Science and Technology, Wuhan, China

**Keywords:** autoimmune encephalitis, anti-AMPA receptor, relapse, brain atrophy, case report

## Abstract

Alpha-amino-3-hydroxy-5-methyl-4-isoxazolepropionic acid (AMPA) receptor encephalitis is a relatively rare anti-neuronal surface antigen autoimmune encephalitis (LE). We described a case of a 47-year-old Chinese man having anti-AMPA receptor limbic encephalitis initially presented with cognitive decline, undetectable antibodies, and normal imaging findings in magnetic resonance image (MRI) and then developed into typical autoimmune limbic encephalitis a few months later with a course of multiple relapses. In addition, we found progressive brain atrophy in our case, which was a rare presentation of LE. This report also summarized the characteristics of nine reported cases of anti-AMPA receptor limbic encephalitis with relapse up to date. This case highlighted that autoimmune limbic encephalitis is an important differential diagnosis for patients with typical symptoms even when the MRI and antibodies are normal, and more attention should be paid to the relapse of anti-AMPA receptor encephalitis.

## Introduction

Autoimmune limbic encephalitis is a neurological disorder characterized by a sub-acute onset of clinical manifestations including mood change, cognition impairment, confusion and seizures, and brain abnormality in the medial temporal lobe on MRI (1). Autoimmune limbic encephalitis is usually accompanied with antibodies, consisting of antibodies against intracellular antigens including Hu, Ma2, and glutamic acid decarboxylase (GAD), and antibodies against cell-surface antigens including synaptic receptors including gamma-aminobutyric acid type B (GABA_B_) receptor, alpha-amino-3-hydroxy-5-methyl-4-isoxazolepropionic acid (AMPA) receptor, and leucine-rich glioma inactivated 1 (LGI 1) (1). The encephalitis associated with antibodies against neuronal cell-surface antigens is more responsive to immunotherapy than those with antibodies against intracellular antigens. The anti-AMPA receptor encephalitis is relatively rare, compared to other autoimmune encephalitides (2). This report describes an anti-AMPA receptor limbic encephalitis case, in which the patient was presented with cognitive decline and without detectable antibodies at first, and developed into typical limbic encephalitis a few months later. The case is unique for its insidious onset with undetectable antibodies at first and the relapses of encephalitis. In addition, this report also summarized the cases of anti-AMPA receptor limbic encephalitis with relapse from January 1, 2000, to December 31, 2020.

## Case Presentation

A 47-year-old man was admitted to our hospital for progressive cognitive decline and apathy for 10 months. History taking revealed no infection, vaccination, or significant weight loss within 6 weeks and no other medical history. His family had no history of auto-immune and hereditary diseases. Further general and neurological examination of the body revealed no abnormalities except for cognitive decline and active tendon reflexes in limbs. The long-term and short-term memory, the ability of calculation, as well as temporal and spatial perception were found to be impaired.

**Figure 1:**
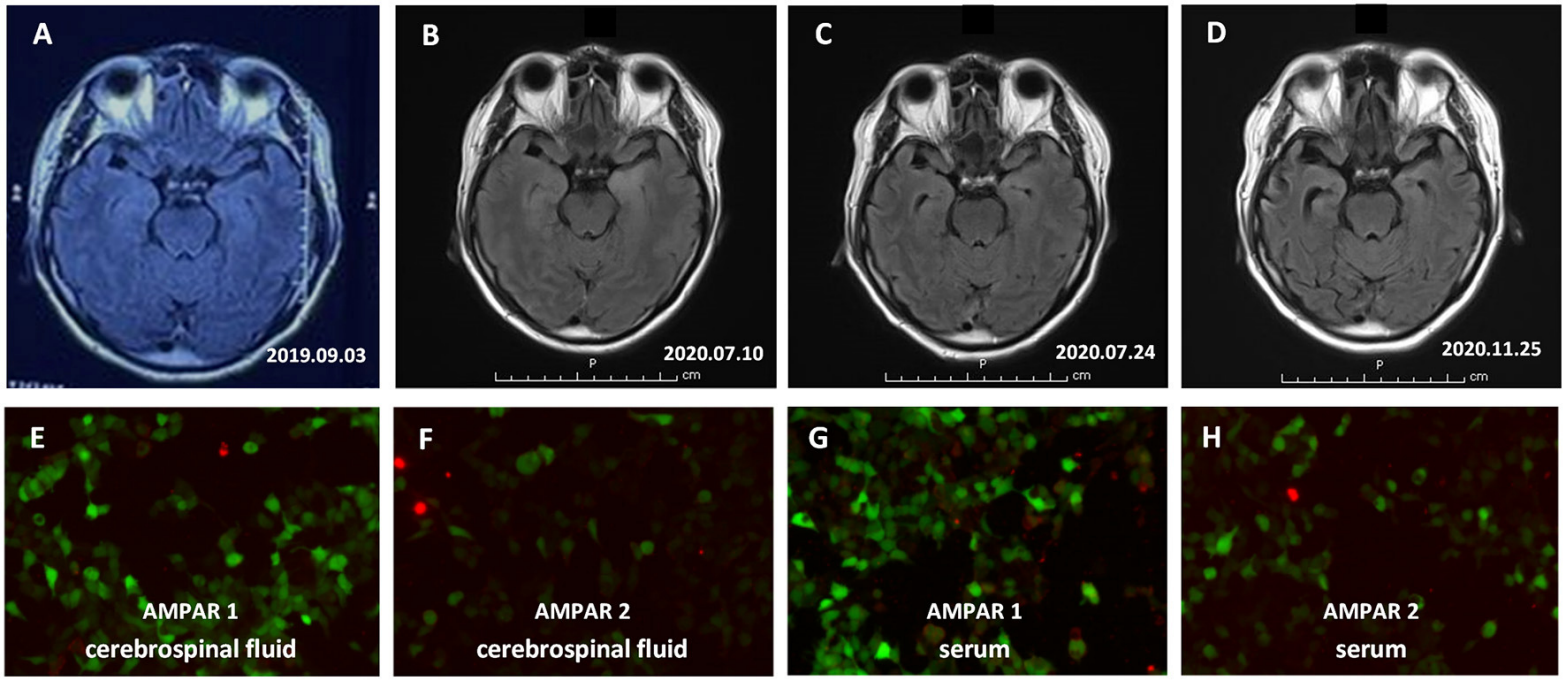
The result of Brain MRI and Anti-alpha-amino-3-hydroxy-5-methyl-4-isoxazolepropionic acid (AMPAR) antibodies in the serum and cerebrospinal fluid (CSF). **(A–D)** Brain MRI showed high signal changes on fluid-attenuated inversion recovery sequences predominantly affecting the bilateral medial temporal lobe combined with some parts of the temporal cortex and progressive brain atrophy. **(A)** At the onset of the disease; **(B)** Onset for more than 10 months; **(C)** After 2 weeks of hormone shock therapy; **(D)** Follow-up for 4 months after discharge. **(E–H)** Anti-AMPAR1 and Anti-AMPAR2 antibodies in the serum and CSF of the patient were positive, as tested by the CBA method. **(E)** Anti-AMPAR1 antibodies in CSF; **(F)** Anti-AMPAR2 antibodies in CSF; **(G)** Anti-AMPAR1 antibodies in serum; **(H)** Anti-AMPAR2 antibodies in serum.

In further elaboration, 10 months ago (September 3, 2019), the patient was admitted to the first hospital for memory loss in 4 days. The symptoms of the patient started with the inability to remember what happened just now, unresponsive, and apathy. But the ability of daily living was not significantly impaired. Intracranial infection and acute cerebrovascular disease were suspected, but initial brain MRI (Figure 1A) and lumbar puncture were normal. The autoantibodies and paraneoplastic antibodies in serum and CSF were not performed in the hospital. The scores of the Geriatric Depression Scale were 12, accordingly, the patient was treated with flupenthixol/melitracen and escitalopram.

About 2 months later (October 28, 2019), he was admitted to the second hospital because of his worsened short-term memory, temporal, and spatial perception. The examination of Mini-Mental State Examination (MMSE) scores was 22/30. Normal findings of routine blood tests included neutrophil/hemoglobin/platelet counts, liver and renal function tests. Other investigations included thyroid function, erythrocyte sedimentation rate, antinuclear antibody, anti-neutrophil cytoplasmic antibody, anti-β2 glycoprotein antibody, anti-cardiolipin antibody, extractable nuclear antigen, complement levels, serum protein electrophoresis, porphyria screen, tumor biomarkers, organic acid, poison screening were within normal limits. Hepatitis B virus serology, HIV serology, treponema pallidum serology, tuberculosis, and CD4 T cell count were negative. CSF analysis was normal, including cells, protein, glucose, oligoclonal bands, herpes simplex virus 1 and 2, varicella-zoster virus, enterovirus, meningococcus, parasites, treponema pallidum. The metagenomic next-generation sequencing (mNGS) was also conducted in the CSF sample but no pathogen (detection ranging from viruses to bacteria, fungi, and parasites) was identified. A repeat MRI brain was normal. Video electroencephalogram (EEG) showed low-amplitude fast waves. Even though the MRI brain and CSF of the patient were normal, autoimmune or paraneoplastic encephalitis was suspected based on the clinical presentation. Further ultrasonography examination of the abdominal organs, thyroid gland, and CT scan of the thorax showed no evidence of malignancy. Screening tests were also negative for autoimmune encephalitis antibody (NMDA-R, CASPR2, AMPA1-R, AMPA2-R, and LGI1), paraneoplastic antibody (Hu, Yo, Ri, PNMA2, CRMP5, and amphiphysin), and myelin-related antibodies (MBP-Ab, MOG-Ab, AQP4-Ab). No special treatment was given due to an unclear diagnosis. While in hospital, he developed rapidly progressive memory deficits, abnormal behavior like teasing children and making funny faces, and personality changes with apathy. He was easy to get lost, in addition to confusion and fatigue. Based on the results of the preliminary physical examination and progression of the disease, autoimmune/viral encephalitis was suspected. Accordingly, the patient was treated with intravenous injection of methylprednisolone 1 g daily, halved every 3 days, and tapered over 12 days and at the same time with antiviral drug acyclovir. Oral prednisolone (70 mg/day) was initiated on day 13, and then gradually tapered to a maintaining dose (10 mg/day). The condition of the patient gradually improved, and he could come back to normal life and work.

In July 2020, the patient was admitted to our hospital for a relapse characterized by memory loss and agitation. Repeat brain MRI showed high signal changes on T2 and fluid-attenuated inversion recovery sequences predominantly affecting the bilateral medial temporal lobe combined with some parts of the temporal cortex (Figure 1B). CSF neural-specific antibodies detection *via* cell-based assay (further details around the laboratory were provided in Supplementary Materials) found antibodies against AMPA1R (titer, 1:1) and AMPA2R (titer, 1:10); serum also showed the presence of anti-AMPA2R (1:32), but anti-AMPA1R was undetected. EEG showed diffuse slow waves. CT scan of thorax/abdomen/pelvis was negative for neoplasia. The diagnosis of autoimmune encephalitis prompted immediate treatment with a second course of methylprednisolone for 12 days (methylprednisolone 1 g daily, halved every 3 days, and tapered over 12 days). A repeat brain MRI showed the increased signal in temporal lobes had improved and there was atrophy of the medial temporal lobe (Figure 1C). The memory of the patient improved, and he could take care of himself with the assistance of family members when discharged.

After 4 months (November 3, 2020), the patient was admitted to our hospital again for a relapse characterized by confusion, dramatic memory deficit, personality changes with irritability, and episodes of aggressive behavior. Antibodies against AMPAR1 and AMPAR2 were detected both in the serum and CSF of the patient. The titer of AMPAR1 is 1:10 in CSF (Figure 1E) and serum (Figure 1G), and the titer of AMPAR2 is 1:100 in CSF (Figure 1F), 1:1000 in serum (Figure 1H). Owing to the recurrence, the patient was treated with intravenous injection of immunoglobulin (IVIG) combined with a repeat course of methylprednisolone and oral immunosuppressive drug (mycophenolate mofetil). He responded well to the treatment with the improvement of abnormal and aggressive behavior. Repeat MRI brain 3 weeks after admission showed significant atrophy of bilateral medial temporal lobe and temporal cortex (Figure 1D). Mycophenolate mofetil (3 g/day) was maintained when discharged. During the follow-up of a half year, he has had no further relapses and returned to work (MMSE 27 and mRS 1).

**Figure 2:**
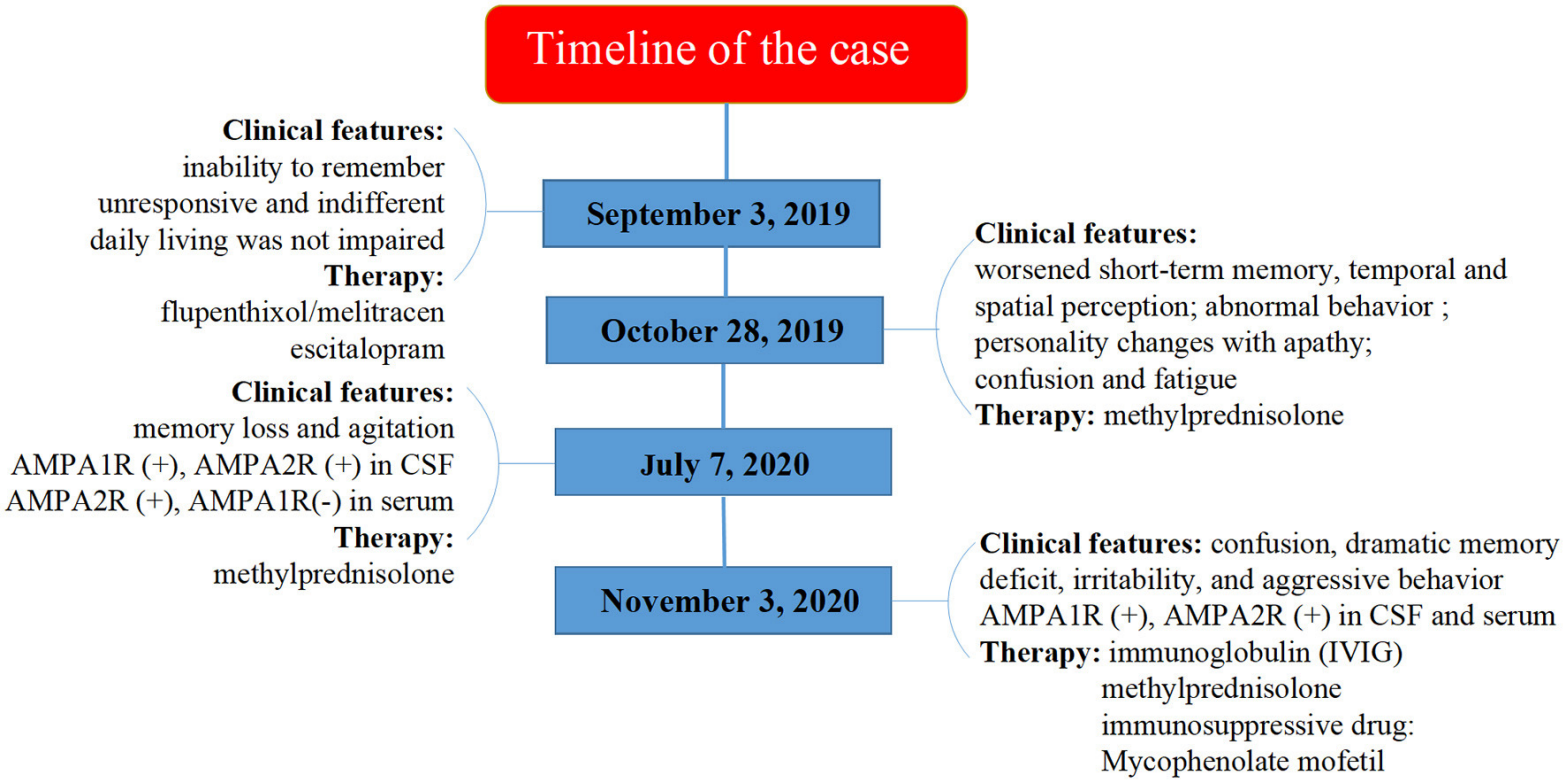
Timeline of the case.

The timeline of the case was summarized in Figure 2.

## Discussion

Anti-AMPA receptor encephalitis can present with partial or total manifestations of typical limbic encephalitis syndromes (including memory loss, confusion, abnormal behavior), diffuse encephalopathy, and other symptoms like seizures and motor deficits (3). It usually affects middle-aged women, has an abnormality in the medial temporal lobes or hippocampus on T2 or FLAIR MRI, and accompanies by tumors involving the lung, thymus, breast, and ovary according to previous reports (3–5). The response to immunotherapy is variable with outcomes ranging from complete neurological recovery, partial recovery, or even death (6).

According to the current criteria (1), the diagnosis items of definite autoimmune limbic encephalitis include four parts: (1) subacute onset of symptoms of memory loss, seizures or psychiatric symptoms, (2) bilateral abnormalities of medial temporal lobes on FLAIR MRI, (3) CSF pleocytosis or EEG abnormity with epileptic or slow-wave activity involving the temporal lobes, (4) exclusion of other possible causes. Although antibodies are not needed in the proposed criteria, the measurement is still very important. When the items of criteria are not fully met, the detection of antibodies can help to establish the diagnosis.

Therefore, the diagnosis of definite autoimmune limbic encephalitis could not be made for the patient at the first attack, because of the normal manifestation of brain MRI and CSF. Through complete testing, alternative causes, such as cerebrovascular disease, intracerebral tumor, and a primary psychiatric disorder, were excluded. Although the antibodies were also not detected, the patient was diagnosed with possible autoimmune limbic encephalitis. Owing to the detection of AMPA receptor antibodies at the second attack and findings on MRI, we made a final diagnosis of autoimmune limbic encephalitis with anti-AMPA receptor antibodies. For patients with CNS syndromes including autoimmune encephalitis and without evidence of MRI and CSF abnormalities, one study showed that the antibodies testing was important for the diagnosis (7). Furthermore, one study also suggested that autoimmune limbic encephalitis can happen without detectable autoantibodies like the situation in our case (8). When the diagnosis is not defined, it is a matter of debate whether the patient should receive immunotherapy. In our case, after discussion with his family, the patient received immunotherapies after 2nd presentation and there was some improvement with the treatment. We didn't find a similar case in anti-AMPA receptor encephalitis in previous reports. Our case suggested the autoimmune limbic encephalitis is an important differential diagnosis for patients with typical symptoms, even when the MRI, CSF, and antibodies are normal.

Another important feature in our case was the relapse of encephalitis. We found 63 cases of anti-AMPA receptor encephalitis with sufficient clinical data and identified 9 cases with relapse of encephalitis (3, 4, 6, 9, 10) (Table 1). The patient median age was 44 years (range 30–87 years); six were female and three were male. The patients presented with typical symptoms of limbic encephalitis, including memory deficit (*n* = 8), abnormal behavior (*n* = 2), confusion (*n* = 6), and many patients presented with seizure (*n* = 6). Six patients had a neoplasm, including thymoma (*n* = 5) and breast cancer (*n* = 1). Five patients had additional antibodies except for the anti-AMPA receptor antibody. The most common abnormality on FLAIR MRI was the increased signal in the medial temporal lobe (*n* = 7) and more than half of patients showed an abnormal EEG. The CSF testing demonstrated an increased cell number or protein level in most patients (*n* = 7). For the treatment of encephalitis, immunotherapies were applied in all cases, including steroids (*n* = 9), intravenous immunoglobulin (*n* = 6), plasma exchange (*n* = 4), azathioprine (*n* = 2), rituximab (*n* = 2), cyclophosphamide (*n* = 1). All the patients responded to the treatment at the first episode with only 3 patients returning to the baseline and having one or more relapses in a median period of 9 months (range 1–101 months). The outcome of these patients varied: one patient was lost to follow-up, one patient died, four patients had residual symptoms, and three patients with almost a full recovery. It was noted that these patients all received immunotherapies but still experienced the relapse of encephalitis a few months later. Complete recovery could occur in some cases, but most patients had residual symptoms or even death. The patient in our case also had a residual memory deficit. Therefore, the relapse of anti-AMPA receptor encephalitis is an important item in the follow-up and should attract the attention of the doctor.

**Table 1:** Summary of nine cases with relapse of AMPA-R antibodies encephalitis.

**Sex/Age**	**Symptoms**					**Tumor**	**Antibody**	MRI^*^	**EEG**	**CSF**	**Treatment**		**Outcome**
	**M**	**A**	**C**	**S**	**other**						**At presentation**	**At relapse**	
F/65(4)	1	1	1		nystagmus	None	AMPA 1	Bilateral medial temporal lobe	normal	cell↑ protein↑ OB +	Steroids PLEX	Steroids IVIg AZA	3 relapses 7 months between presentation and last relapse First episode: returned to baseline; After relapse: residual behavioral problem and memory deficit
F/44(4)	1		1	1	combativeness nystagmus	thymoma	AMPA 2 ANA dsDNA ACA	medial temporal lobe (Right; Left)	diffuse theta activity; episodes of epileptic activity in left temporal lobe	cell↑ protein↑ OB -	Tumor removal Steroids IVIg AZA	Steroids IVIg AZA	3 relapses 101 months between presentation and last relapse First episode: returned to baseline; After relapse: residua memory deficit
M/38(4)	1		1	1	agitation	thymoma	AMPA 2 GAD	right medial and lateral temporal lobe, right frontal, left insular and occipital regions	NA	cell↑ protein↑ OB +	Tumor removal Radiation PLEX Steroids IVIg		1 relapse 60 months between presentation and last relapse First episode: returned to baseline; After relapse: residual memory deficit; steroid dependent muscle spasms and rigidity
F/87(4)	1			1	disorientation	None	AMPA 1 ANA	Bilateral medial temporal lobe	Diffuse slow activity, delta activity in anterior frontotemporal area	protein↑	Steroids		1 relapse 16 months between presentation and last relapse First episode: partial improvement; After relapse: death
F/61(4)	1			1	decreased level of consciousness	breast cancer	AMPA 2	Normal	Theta activity in posterior temporal regions	cell↑ protein↑	Steroids	Tumor removal PLEX Steroids	1 relapse 9 months between presentation and last relapse First episode: response to treatment; After relapse: residual behavioral problem and memory deficit
M/44(6)	1				dystonia	thymoma	AMPA CRMP-5	Bilateral hippocampal	general slowing	Normal	Steroids IVIg rituximab	Steroids IVIg rituximab	1 relapse 3.5 weeks between presentation and last relapse First episode: response to treatment and discharge After relapse: mRS 0 and return to work
M/30(9)			1	1	difficulty walking	thymoma	AMPA VGKC NMDA	left caudate nucleus	NA	cell↑	Tumor removal Steroids IVIg PLEX CTX	Tumor removal Rituximab CTX	1 relapse 1 month between presentation and last relapse First episode: response to treatment and discharge After relapse: significant improvement
F/34(10)	1		1		agitation gait disturbance	thymoma	AMPA	left caudate nucleus; right insula and right temporal lobe	focal epileptic discharges at the left temporal-parietal region	Normal	Tumor removal Steroids	Steroids	1 relapse 47 months between presentation and last relapse First episode: residual depressive symptom; After relapse: MMSE 29
F/72(3)	1	1	1	1		None	AMPA	Bilateral medial temporal lobe	general slowing	cell↑ protein↑	Steroids IVIg		1 relapse 2 months between presentation and last relapse First episode: partial response to treatment; After relapse: lost to follow-up

*M, memory deficits; A, abnormal behavior; C, confusion; S, seizure; MRI, magnetic resonance image; EEG, electroencephalogram; CSF, Cerebrospinal fluid; AMPA, alpha-amino-3-hydroxy-5-methyl-4-isoxazolepropionic acid; ANA, antinuclear antibody; dsDNA, double stranded DNA; ACA, anti-cardiolipin antibodies; GAD, glutamic acid decarboxylase; CRMP-5, collapsing response mediator protein-5; VGKC, voltage-gated potassium channel; NMDA, N-methyl-D-aspartate; OB, oligoclonal bands; NA, not applicable; PLEX, plasma exchange; IVIg, intravenous immunoglobulin; AZA, azathioprine; CTX, cyclophosphamide; mRS, modified Rankin Scale; MMSE, Mini-Mental State Examination*.

* *Brain regions with increased signal on FLAIR MRI are listed*.

Furthermore, serial brain MRI of the patient clearly revealed progressive brain atrophy, which was predominantly in the medial temporal lobes and temporal cortex. Brain atrophy is a rare presentation of LE and only reported in one case up to date (11). In our case, the patient had no hypoxic event and status epilepticus. Two possible mechanisms of brain atrophy may be considered: one is the use of steroid treatment and the other is the internalization of AMPA receptors. It had been reported that brain atrophy is associated with the use of corticosteroids in the previous study (12). High-dose corticosteroids may contribute to reversible short-term loss of volume, while chronic low doses of corticosteroids may induce irreversible loss of tissue through steroid-induced protein catabolism (12). Our patients received high-dose intravenous methylprednisolone during the acute and relapsed stage of the disease and low-dose oral prednisone/methylprednisolone after discharge. Therefore, we cannot exclude a potential effect of corticosteroids treatment. Moreover, receptor internalization may be another possible mechanism (4, 13). It is supposed that the internalization of AMPA receptors might cause synaptic silencing, dendrite degeneration, or neuronal loss (14). It was suspected that the progressive brain atrophy of our patients may be related to a potential effect of AMPAR immune response. Despite the presence of progressive brain atrophy, the gradual function recovery of our patient does not necessarily indicate a poor clinical outcome, which needs a long-term follow-up to confirm.

## Data Availability Statement

The original contributions presented in the study are included in the article/Supplementary Material, further inquiries can be directed to the corresponding author.

## Ethics Statement

Written informed consent was obtained from the participant for the publication of this case report. Written informed consent was obtained from the individual(s) for the publication of any potentially identifiable images or data included in this article.

## Author Contributions

YF and HH designed the study, drafted, and revised the manuscript. YF mainly drafted the part of abstract and case presentation. HH performed the part of introduction and discussion. DP provided input and suggestions into the study. HH had full access to the data in the study and takes responsibility for the integrity of the data and accuracy of the case. All authors reviewed and approved the final manuscript.

## Funding

The study was supported by the National Natural Science Foundation of China (81571206).

## Conflict of Interest

The authors declare that the research was conducted in the absence of any commercial or financial relationships that could be construed as a potential conflict of interest.

## Publisher's Note

All claims expressed in this article are solely those of the authors and do not necessarily represent those of their affiliated organizations, or those of the publisher, the editors and the reviewers. Any product that may be evaluated in this article, or claim that may be made by its manufacturer, is not guaranteed or endorsed by the publisher.

## Abbreviations

AMPA: Alpha-amino-3-hydroxy-5-methyl-4-isoxazolepropionic acid
LE: autoimmune encephalitis
MRI: magnetic resonance image
GAD: glutamic acid decarboxylase
GABAB: gamma-aminobutyric acid type B
LGI 1: leucine-rich glioma inactivated 1
MMSE: Mini-Mental State Examination
EEG: electroencephalogram
CSF: Cerebrospinal fluid
IVIG: Immunoglobulin
OCB: oligoclonal bands.
