# Supplementary material for: Recurrent Anti-AMPA Receptor Limbic Encephalitis: A Case Report and Literature Review
Source: Front Neurol. 2021 Dec 6;12:735983. doi: 10.3389/fneur.2021.735983 (PMC8690251; doi:10.3389/fneur.2021.735983)
Supplement: Supplementary file 1 [file Table_1.pdf]

Supplementary materials

The methodology utilized for the testing of the autoimmune encephalitis antibodies: Cell-based assays

Indirect immunofluorescence assay (IFT) based on cell-based assays was used for detecting antibodies to neuronal surface antigens. Human embryonic kidney cells (HEK293) were transiently transfected with cDNA of the antigen of interest (together with Green Fluorescent Protein) using the kit (Shaanxi MYBiotech Co., Ltd), including anti-NMDAR antibody, anti-LGI1 antibody, anti-CASPR2 antibody, anti-AMPAR antibody, anti-GABabR antibody. Using the kit recommended testing steps: Serum [diluted with 1：10 phosphate buffer (PBS)] and cerebrospinal fluid (120μL each) to be tested were dropped to the reaction zone with the sample. Then, the transfected cells are incubated with the sample at room temperature for 30 min. Anti-human IgG labeled with 120μL FITC was added and incubated at room temperature for 30 min, then washed with PBS and observed under fluorescence microscope. The result was as follows:

The report for Autoimmune encephalitis antibody			
Type of antibody	Result	The method for detection	Reference range
NMDAR	negative	Cell-based assays	negative
LGI1	negative	Cell-based assays	negative
CASPR2	negative	Cell-based assays	negative
GABABR	negative	Cell-based assays	negative
AMPA1R	positive (1:10+)	Cell-based assays	negative
AMPA2R	positive (1:100+)	Cell-based assays	negative

Contribution to the field statement

Alpha-amino-3-hydroxy-5-methyl-4-isoxazolepropionic acid (AMPA) receptor encephalitis is a relatively rare anti-neuronal surface antigen autoimmune encephalitis (LE). We described a 47 year-old Chinese man case of anti-AMPA receptor limbic encephalitis. The case is unique for its insidious onset with undetectable antibodies and normal imaging findings in magnetic resonance image (MRI) at first and then developed into typical autoimmune limbic encephalitis a few months later with a course of multiple relapses and a tortured treatment. In addition, we found a progressive brain atrophy in our case, which was a rare presentation of LE. This report also summarized the characteristics of nine reported cases of anti-AMPA receptor limbic encephalitis with relapse up to date. This case highlights that autoimmune limbic encephalitis is an important differential diagnosis for patients with typical symptoms even when the MRI and antibodies are normal and more attention should be paid for the relapse of anti-AMPA receptor encephalitis.

Informed Consent Form for Publication of Case Reports

English version：

Dear Fen,

We are very sorry to inform you that you were considered to be diagnosed as AMPA receptor encephalitis according to your clinical data, which is a relatively rare anti-neuronal surface antigen autoimmune encephalitis.

During the course, although you had repeated recurrence of your disease and developed to brain atrophy. You responsed well to our treatment and gradually improved finally. This type of case is rarely reported in the world. We hope to publish relevant content to attract the doctor’s attention for this type of disease. The published articles will show your entire course of the disease and the outcome of treatment without disclosure of your private information. We sincerely hope to get your consent. If you agree, you can sign this informed consent form.

Yours sincerely,

Yuanyuan Fang

November 27, 2020

Chinese version：

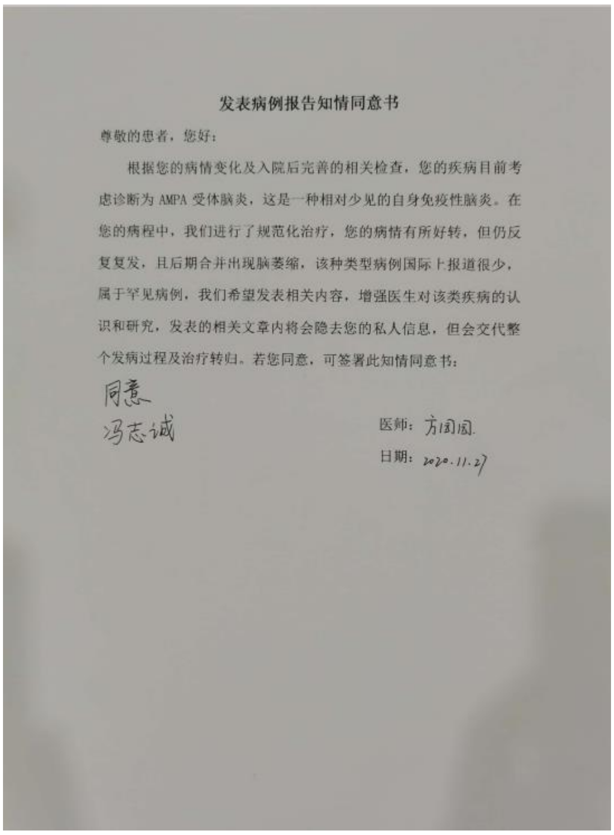

发表病例报告知情同意书

尊敬的患者，您好：

根据您的病情变化及入院后完善的相关检查，您的疾病目前考虑诊断为 AMPA 受体脑炎，这是一种相对少见的自身免疫性脑炎。在您的病程中，我们进行了规范化治疗，您的病情有所好转，但仍反复复发，且后期合并出现脑萎缩，该种类型病例国际上报道很少，属于罕见病例，我们希望发表相关内容，增强医生对该类疾病的认识和研究，发表的相关文章内将会隐去您的私人信息，但会交代整个发病过程及治疗转归。若您同意，可签署此知情同意书：

同意

冯志诚

医师：方圆圆

日期：2020.11.27

CARE case report guidelines

CARE Checklist of information to include when writing a case report

(cc) BY-NC-ND

Topic	Item	Checklist item description	Reported on Line
**Title**	1	The diagnosis or intervention of primary focus followed by the words "case report"	page 1
**Key Words**	2	2 to 5 key words that identify diagnoses or interventions in this case report, including "case report"	Lines 16-17, page 1
**Abstract (no references)**	3a	Introduction: What is unique about this case and what does it add to the scientific literature?	Lines 2-4, page 1
	3b	Main symptoms and/or important clinical findings	Lines 4-11, page 1
	3c	The main diagnoses, therapeutic interventions, and outcomes	Lines 4-11, page 1
	3d	Conclusion—What is the main "take-away" lesson(s) from this case?	Lines 11-14, page 1
**Introduction**	4	One or two paragraphs summarizing why this case is unique (**may include references**)	Lines 1-19, page 2
**Patient Information**	5a	De-identified patient specific information.	Lines 22, page 2
	5b	Primary concerns and symptoms of the patient.	Lines 22-23/29-31, page 2
	5c	Medical, family, and psycho-social history including relevant genetic information	Lines 23-25, page 2
	5d	Relevant past interventions with outcomes	Lines 32-33, page 2
**Clinical Findings**	6	Describe significant physical examination (PE) and important clinical findings.	Lines 25-28, page 2
**Timeline**	7	Historical and current information from this episode of care organized as a timeline	Lines 29/34, p2; Lines 17/29, p3
**Diagnostic Assessment**	8a	Diagnostic testing (such as PE, laboratory testing, imaging, surveys).	Lines 35-44, p2; Lines 1-6/18-23, p3
	8b	Diagnostic challenges (such as access to testing, financial, or cultural)	Lines 6-9, page 3
	8c	Diagnosis (including other diagnoses considered)	Lines 9-11, page 3
	8d	Prognosis (such as staging in oncology) where applicable	Lines 15-16,25-28, page 3
**Therapeutic Intervention**	9a	Types of therapeutic intervention (such as pharmacologic, surgical, preventive, self-care)	Lines 11-15, page 3
	9b	Administration of therapeutic intervention (such as dosage, strength, duration)	Lines 11-15, page 3
	9c	Changes in therapeutic intervention (with rationale)	Lines 34-37, page 3
**Follow-up and Outcomes**	10a	Clinician and patient-assessed outcomes (if available)	Lines 40-41, page 3
	10b	Important follow-up diagnostic and other test results	Lines 38-39, page 3
	10c	Intervention adherence and tolerability (How was this assessed?)	None
	10d	Adverse and unanticipated events	None
**Discussion**	11a	A scientific discussion of the strengths AND limitations associated with this case report	Lines 33-36,38, p3; Lines 22-23, p5
	11b	Discussion of the relevant medical literature **with references**.	Lines 27-30,38-44, p3; Lines1-17, 21,p5
	11c	The scientific rationale for any conclusions (including assessment of possible causes)	Lines 22-34, page 5
	11d	The primary "take-away" lessons of this case report (without references) in a one paragraph conclusion	None
**Patient Perspective**	12	The patient should share their perspective in one to two paragraphs on the treatment(s) they received	Supplementary material
**Informed Consent**	13	Did the patient give informed consent? Please provide if requested	Yes ■ No ☐
